# The Effect of Streaming Potential and Viscous Dissipation in the Heat Transfer Characteristics of Power-Law Nanofluid Flow in a Rectangular Microchannel

**DOI:** 10.3390/mi11040421

**Published:** 2020-04-17

**Authors:** Shuyan Deng, Quan An, Mingying Li

**Affiliations:** 1 Institute of Architecture and Civil Engineering, Guangdong University of Petrochemical Technology, Maoming 525000, China; dshy530@sina.cn; 2 Earthquake Administration of Inner Mongolia Autonomous Region, Hohhot 010021, China; quanan818@sina.cn

**Keywords:** power-law nanofluid, heat transfer, streaming potential, electrokinetic flow, Nusselt number

## Abstract

The non-Newtonian nanofluid flow becomes increasingly important in enhancing the thermal management efficiency of microscale devices and in promoting the exploration of the thermal-electric energy conversion process. The effect of streaming potential and viscous dissipation in the heat transfer characteristics of power-law nanofluid electrokinetic flow in a rectangular microchannel has been investigated to assist in the development of an energy harvesting system. The electroviscous effect caused by the streaming potential influences the hydrodynamical and thermal characteristics of flow. With the change in constitutive behavior of power-law nanofluid, the viscous dissipation effect is considered. The Poisson–Boltzmann equation, the modified Cauchy momentum equation, and the energy equation were solved. The temperature and heat transfer rate were analytically expressed for Newtonian nanofluid and numerically obtained for power-law nanofluid. The interactive influence of streaming potential, viscous dissipation, and hydrodynamical features of power-law nanofluid on the heat transfer characteristics were studied. The presence of streaming potential tends to reduce the dimensionless bulk mean temperature. The introduction of nanoparticles augments dimensionless temperature difference between channel wall and bulk flow, which decreases the heat transfer rate. The shear thinning nanofluid is more sensitive to the above effects. The temperature is a weak function of the flow behavior index.

## 1. Introduction

The great advancement of microfabrication technologies has led to the wide application of lab-on-chip-based microsystems for chemical and biomedical analysis [1], and heat sinks for electronic cooling [2]. Importantly, the flow inside the microchannel is not simply the scaled-down version of a conventional macrochannel flow, which shows distinct behaviors such as the electrokinetic phenomena and has inspired the development of fluidic transportation technologies [3]. The interaction of the microchannel wall with electrolyte solution renders the wall surface charged. It is responsible for the rearrangement of ions in solution and the formation of electric double layer (EDL) composing of the immobile compact layer with ions close to the wall and the mobile diffuse layer with counter ions. The typical EDL thickness is comparable with the microchannel dimension on the order of dozens of microns or less. As an electric potential is applied tangentially along the microchannel, the migration of co-ions within EDL drags bulk liquid, resulting in the so-called electroosmotic flow (EOF) [4]. The recent focus on the EOF shifts from the transport characteristics of EOF in different microgeometries and for various working liquids [5,6,7,8,9,10] to rotating flows [11,12], the effects of external magnetic field [13], and pressure gradient [14] or two-layer EOF [15].

Within a pressure-driven flow (PDF), the repelled co-ions in EDL are carried streamwise, inducing the streaming current and further streaming potential. For balance, the conducting current is generated under the streaming potential, and the co-ions flow back, pulling the liquid molecules in the opposite direction to the PDF because of viscous force. Consequently, the flow rate is reduced, being similar to driving a liquid with a higher apparent viscosity. This electroviscous effect is also known as the streaming potential effect, and the PDF with such an effect is one type of electrokinetic flow [16] studied in the present study. Furthermore, due to the frequent operation of biofluids in microfluidic devices, it is inevitable to provide a thorough understanding on the features of non-Newtonian electrokinetic flow. The power-law model proposed by Das and Charkraborty [5] has received great attention because of the concise expression for non-Newtonian working liquids. A more comprehensive review on the hydrodynamical transport of electrokinetic flow can be found in [17,18]. On the other hand, the application of cooling system in MEMS has generated a number of pieces of research on the heat transfer of electrokinetic flow [16,19,20]. The combined effects of streaming potential and wall slip on the heat transfer in PDF were investigated where the viscous dissipation and Joule heating were neglected [21]. Matin extended the model above by taking into account viscous dissipation and Joule heating [22]. They came to the conclusion that the streaming potential plays a remarkable role in thermal transport, which depends on the zeta potential, Brinkman number, and electrokinetic width. A comprehensive analysis shows that in addition to the investigation on EOF in [23], the study on the thermal transport characteristics of power-law fluid flow including Newtonian flow is mainly confined to parallel microchannels [24,25,26,27] and microcapillaries [9,20].

The temperature rise resulting from electrokinetic phenomenon exerts influence on microscale flows. To efficiently remove the internal heat and remain an environment with uniform temperature, the addition of nanoparticle with a diameter of 1 to 100 nm into the bulk fluid [28,29] becomes increasingly popular. It is called nanofluid where the nanoparticles are metal oxide such as AI_2_O_3_ with higher thermal conductivity, and the bulk fluid is called base fluid. The thermal behavior of mixed convection flow for power-law nanofluid is studied in [30,31], in which, Carboxymethyl cellulose-water (CMC-water) with aluminum oxide (AI_2_O_3_) and Polyvinyl chloride (PVC) solution with Cu are represented by power-law nanofluid model. In addition, the researchers investigated the transient thermal characteristics of Newtonian nanofluid EOF [29] and PDF with streaming potential effect [32]. Since non-Newtonian fluids have been manipulated in micropumps and microreactors, the heat transfer characteristics of EOF for power-law nanofluid in a parallel plate microchannel were investigated by analytically solving the one-dimensional momentum equation and energy equation [33]. Due to the variety of microchannels applied in microdevices, the heat transfer of EOF of power-law nanofluid in a rectangular microchannel was discussed by Deng [34] based on the Brinkman model [35]. 

An up-to-date literature review shows that the thermal behaviors of electrokinetic flow with the interactive effects of streaming potential, nanoparticles and power-law constitutive relation have been rarely investigated. It can be attributed to the highly nonlinear constitutive relation of power-law nanofluid, which makes the coupling between hydrodynamical field and temperature field more complicated. More recently, combining the streaming potential effect and temperature gradient over the porous carbon film, Liu et al. developed a thermal-electric nanogenerator based on a new type of thermal-electric conversion process [36]. The unique characteristics of such a conversion process provide a promising and cost-effective way to utilize low-temperature gradient and develop an energy harvesting system for microelectronics. Therefore, due to the potential application of combined streaming potential and non-Newtonian nanofluid, it is desired to provide an insight to the multiple physical phenomena that show how the physical processing interacts with each other. Since the viscous dissipation is associated with the viscosity of power-law nanofluid, it is inevitable to consider the corresponding contributions in a temperature field. This paper aims to investigate the effect of streaming potential and viscous dissipation in thermal transport characteristics of fully-developed PDF for power-law nanofluid in a rectangular microchannel. 

## 2. Mathematical Modeling 

**Figure 1:**
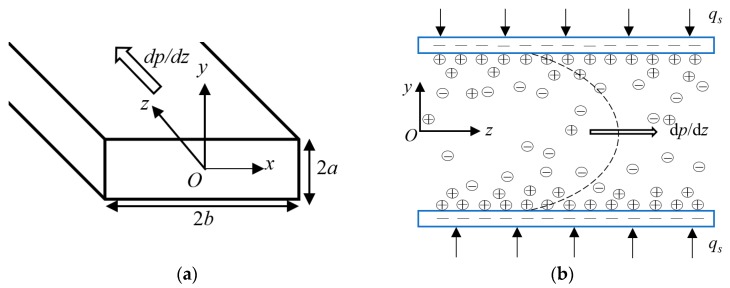
The sketch of the rectangular microchannel with height 2*a* and width 2*b*. (**a**) Three-dimensional sketch, (**b**) Two-dimensional sketch.

A laminar, fully developed, and incompressible PDF of power-law nanofluid with streaming potential effect through a rectangular microchannel of width 2*a* and height 2*b* is considered, as sketched in Figure 1. The flow is driven by the pressure gradient d*p*/d*z*. The channel surface is subject to a uniform zeta potential *ξ* (*ξ* ≤ 0.025 V) and constant heat flux *q_s_*. It is also assumed that the electrolyte solution considered here is ionically symmetric, EDLs on the solid surface do not overlap, and the constant thermophysical properties are applied due to the low temperature variation. As an axial pressure gradient is imposed on the rectangular microchannel, the PDF with the streaming potential effect, namely, the electrokinetic flow occurs. Since the channel length is much longer than the width and height, the electric potential and velocity distribution can be seen as functions of *x* and *y*. Because of the symmetry, the volumetric domain *Ω* for the mathematical modeling below is confined to a quarter cross-section of the rectangular microchannel.

### 2.1. Electric Potential Field

According to the expression of volumetric net charge density, namely, *ρ_e_* = *−*2*z_v_en*_0_sinh[*z_v_eψ*/(*k_B_T_a_*)], and with the assumptions above, the Poisson–Boltzmann (P–B) equation governing the electric potential *ψ* and the corresponding boundary conditions can be given as

(1) $$\frac{\partial^{2}\psi }{\partial x^{2}}+\frac{\partial^{2}\psi }{\partial y^{2}}=-\frac{\rho_{e}}{\varepsilon \varepsilon_{0}}$$

*∂ψ/∂x*|_*x*=0_ = 0, *∂ψ/∂y*|_*y*=0_ = 0, *ψ*|_*x*=*b*_ = *ξ*, *ψ*|_*y*=*a*_ = *ξ* (2)

Several dimensionless variables are introduced for the simplification of the mathematical model: $\bar{x}=x/D_{h}$, $\bar{y}=y/D_{h}$, $\bar{\xi }=z_{v}e\xi /(k_{B}T_{a})$, the characteristic width of microchannel *D_h_ =* 4*ab*/(*a+b*), the reciprocal of EDL thickness κ *=* [2*z_v_e*^2^*n*_0_/(*εε*_0_*k_B_T_a_*)]^1/2^, and the electrokinetic width $K=\kappa D_{h}$ where *ε* is the relative permittivity, *ε*_0_ denotes the permittivity in vacuum, *z_v_* is the valence of ions, *e* is the elementary charge, *k_B_* is the Boltzmann constant, *n*_0_ is the ionic number concentration of the bulk at neutral condition, and *T_a_* is the absolute temperature, respectively. As a result, the dimensionless P–B equation under Debye–Hückel approximation $\left(\sinh\bar{\psi }\approx \bar{\psi }\right)$ [5] and the corresponding boundary conditions are obtained as

(3) $$\frac{\partial^{2}\bar{\psi }}{\partial {\bar{x}}^{2}}+\frac{\partial^{2}\bar{\psi }}{\partial {\bar{y}}^{2}}=K^{2}\bar{\psi }$$

(4) $$\partial \bar{\psi }/\partial \bar{x}{|}_{\bar{x}=0}=0,\partial \bar{\psi }/\partial \bar{y}{|}_{\bar{y}=0}=0,\;\bar{\psi }{|}_{\bar{x}=b/D_{h}}=\bar{\xi },\bar{\psi }{|}_{\bar{y}=a/D_{h}}=\bar{\xi }.$$

### 2.2. Hydrodynamic Field

The assumptions associated with the electrokinetic flow above yield that the velocity components have to satisfy *w* = *w*(x,y) and *u* = *v* = 0, where *u*, *v,* and *w* are the velocity components in *x*-, *y*-, and *z*-direction, respectively. Hence, the material derivative of velocity vanishes. In the meantime, the continuity equation is satisfied. Therefore, the modified Cauchy momentum equation governing the hydrodynamic field of the electrokinetic flow of power-law nanofluid becomes

(5) $$\frac{nm}{{\left(1-\phi \right)}^{5/2}}\left({\left|\frac{\partial w}{\partial x}\right|}^{n-1}\frac{\partial^{2}w}{\partial x^{2}}+{\left|\frac{\partial w}{\partial y}\right|}^{n-1}\frac{\partial^{2}w}{\partial y^{2}}\right)-(\frac{dp}{dz}+E_{s}\rho_{e})=0$$

The boundary conditions are acquired, where the no-slip condition on the wall surface is applied:

(6) $$\partial w/\partial x{|}_{x=0}=0,\;\partial w/\partial y{|}_{y=0}=0,\;w{|}_{x=b}=0,\;w{|}_{y=a}=0$$

Here *n* represents the flow behavior index. *n* < 1 corresponds to shear thinning nanofluid, *n* = 1 corresponds to Newtonian nanofluid and *n* > 1 is for shear thickening nanofluid. Plus, the first term in left hand side (LHS) of Equation (5) indicates the shear stress of power-law nanofluid, derived from the stress tensor $\dot{\tau }=2\mu_{eff}\dot{\gamma }\vec{e}$, the strain rate tensor $\dot{\gamma }={\left(2e_{kl}e_{kl}\right)}^{1/2}$, and *e_ij_ =* [(*∂w_i_/∂x_j_*)*+*(*∂w_j_/∂x_i_*)]/2 [7,17]. According to the model developed by Brinkman [35], the effective viscosity of the power-law nanofluid *μ_eff_ = μ_f_*/(1*−ϕ*)^5/2^ [31]. The viscosity of base fluid is expressed as *μ_f_ = m*(*|∂w/∂x|^n−^*^1^*, |∂w/∂y|^n−^*^1^) based on the power-law model and the assumptions of PDF, which shows dependence on the strain rate, flow consistency index *m* of dimension [N·m^−2^·s*^n^*], and the flow behavior index *n* [7,17]. The second term in the LHS of Equation (5) denotes the axial pressure gradient, namely, the driving force of PDF. The third term in the LHS of Equation (5) represents the resistance force arising from the presence of streaming potential, namely the measurement of the streaming potential effect, where *E_s_* is the strength of induced electric field in EDL.

Firstly, the streaming current *I_s_*(*t*) along the flow direction is expressed as

(7) $$I_{s}(t)=\int_{0}^{b}\int_{0}^{a}w(x,y,t)\rho_{e}(x,y)dxdy$$

The streaming electric potential caused by the migration of ions in streamwise in turn induces the so-called conducting current, which is opposite to the direction of PDF, namely, *I_c_*(*t*) = *σA_s_E_s_*(*t*) where *σ* is the total electrical conductivity of the electrolyte solution and solid surface, and *A_s_* represents the rectangular cross-sectional area. Based on the ionic net current equilibrium condition in the rectangular microchannel, one has *I_s_*(*t*) *+ I_c_*(*t*) *=* 0, and thus, the strength of induced electric field is expressed as

(8) $$E_{s}(t)=\frac{1}{\sigma ab}\int_{0}^{b}\int_{0}^{a}w(x,y,t)\rho_{e}(x,y)dxdy$$

The following dimensionless group are introduced: $\bar{w}=w/W$, Re0=ρWDh/μ0, dp¯/dz¯=dp/dz⋅DhRe0/(ρW2),E¯s=EsDhRe0/ξ,$G_{1}=2z_{v}en_{0}\xi /(\rho W^{2})$ expressing the ratio of electric energy to the mechanical kinetic energy, $G_{2}=2z_{v}en_{0}D_{h}W/(\sigma \xi )$ denoting the ratio of the streaming current to the conducting current, A1=Re0Dh2G2/(ab), $A_{2}=4D_{h}{}^{2}G_{1}/(ab)$ where *μ*_0_ denotes the viscosity coefficient of Newtonian fluid, one eventually has the dimensionless modified Cauchy momentum equation and the strength of induced electric field

(9) $$\frac{nm}{\mu_{0}{\left(1-\phi \right)}^{5/2}}{\left(\frac{W}{D_{h}}\right)}^{n-1}\left({\left|\frac{\partial \bar{w}}{\partial \bar{x}}\right|}^{n-1}\frac{\partial^{2}\bar{w}}{\partial {\bar{x}}^{2}}+{\left|\frac{\partial \bar{w}}{\partial \bar{y}}\right|}^{n-1}\frac{\partial^{2}\bar{w}}{\partial {\bar{y}}^{2}}\right)-(\frac{d\bar{p}}{dz}+G_{1}{\bar{E}}_{s}\bar{\psi })=0$$

(10) $$\partial \bar{w}/\partial \bar{x}{|}_{\bar{x}=0}=0,\;\partial \bar{w}/\partial \bar{y}{|}_{\bar{y}=0}=0,\;\bar{w}{|}_{\bar{x}=b/D_{h}}=0,\bar{w}{|}_{\bar{y}=a/D_{h}}=0$$

(11) E¯s=Re0Dh2G2ab∫0a/Dh∫0b/Dhψ¯(x¯,y¯)w¯(x¯,y¯)dx¯dy¯

### 2.3. Thermal Field

The energy equation governing the thermal field of electrokinetic flow with consideration of viscous dissipation and Joule heating effect is

(12) $${\left(\rho c_{p}\right)}_{eff}w\frac{\partial T}{\partial z}=k_{eff}\nabla^{2}T+\Phi +\sigma E_{s}{}^{2}$$

where the first term in the right hand side (RHS) of Equation (12) implies the conduction heat generation, the second term in the RHS of Equation (12) has the form of $\Phi =\frac{m}{{\left(1-\phi \right)}^{5/2}}\left[{\left|\frac{\partial w}{\partial x}\right|}^{n-1}{\left(\frac{\partial w}{\partial x}\right)}^{2}+{\left|\frac{\partial w}{\partial y}\right|}^{n-1}{\left(\frac{\partial w}{\partial y}\right)}^{2}\right]$, which stands for the volumetric heat generated from the viscous dissipation, the last term in the RHS of Equation (12) denotes the Joule heating generated from the ohmic resistance of the electrolyte solution. Besides, ${\left(\rho c_{p}\right)}_{eff}=\phi {\left(\rho c_{p}\right)}_{s}+(1-\phi ){\left(\rho c_{p}\right)}_{f}$ and $k_{eff}=\frac{k_{s}+2k_{f}+2(k_{s}-k_{f}){\left(1+\omega \right)}^{3}\phi }{k_{s}+2k_{f}-2(k_{s}-k_{f}){\left(1+\omega \right)}^{3}\phi }k_{f}$ [37,38]. *T* represents temperature field, *ω* implies the ratio of the nanolayer thickness to the original particle radius. *k* and (*ρc_p_*) denote thermal conductivity and heat capacity of power-law nanofluid at the reference pressure, respectively. The subscripts *s*, *f*, and *eff* stand for the solid nanoparticles, base fluid, and nanofluid, respectively. The boundary conditions that Equation (12) obeys are

(13) $$\partial T/\partial x{|}_{x=0}=0,\partial T/\partial y{|}_{y=0}=0,\;\partial T/\partial x{|}_{x=b}=q_{s}/k_{eff},\;\partial T/\partial y{|}_{y=a}=q_{s}/k_{eff}\;(\mathrm{or}\;T{|}_{y=a}=T_{w}(z))$$

When considering a thermally fully developed PDF with streaming potential effect, the thermal field satisfies *∂*[(*T-T_w_*)/(*T_m_-T_w_*)]/*∂z* = 0 where *T_m_* represents the mean temperature over the cross-sectional area of the rectangular microchannel, and *T_w_* stands for the wall temperature, which varies along the axial direction due to the axial thermal conduction on the wall. Consequently, when applying the constant wall heat flux, i.e., *q_s_* = *const.*, it is derived that *∂T*/*∂z* = d*T_w_*/d*z* = d*T_m_*/d*z* = *const* and *∂*^2^*T*/*∂z*^2^ = 0. Therefore, Equation (12) falls into the following simplified form

(14) $${\left(\rho c_{p}\right)}_{eff}w\frac{dT_{m}}{dz}=k_{eff}\left(\frac{\partial^{2}T}{\partial x^{2}}+\frac{\partial^{2}T}{\partial y^{2}}\right)+\Phi +\sigma E_{s}{}^{2}$$

Imposing the global energy balance condition over an elemental control volume on a length of duct d*z* produces

$$\frac{dT_{m}}{dz}=\frac{q_{s}(a+b)+ab\sigma E_{s}{}^{2}}{{\left(\rho c_{p}\right)}_{eff}abw_{m}}+\frac{m}{{\left(1-\phi \right)}^{5/2}{\left(\rho c_{p}\right)}_{eff}abw_{m}}\int_{0}^{a}\int_{0}^{b}\left[{\left|\frac{\partial w}{\partial x}\right|}^{n-1}{\left(\frac{\partial w}{\partial x}\right)}^{2}+{\left|\frac{\partial w}{\partial y}\right|}^{n-1}{\left(\frac{\partial w}{\partial y}\right)}^{2}\right]dxdy.$$

With the dimensionless temperature $\bar{T}=k_{f}(T-T_{w})/(q_{s}D_{h})$, the wall heat flux *q_s_*, the dimensionless mean velocity ${\bar{w}}_{m}$ and the Joule heating parameter $S=\sigma E_{s}{}^{2}D_{h}/q_{s}$ representing the ratio of Joule heating to the heat flux from the wall surface, one obtains the dimensionless version of Equation (14)

(15) $$\frac{\partial^{2}\bar{T}}{\partial {\bar{x}}^{2}}+\frac{\partial^{2}\bar{T}}{\partial {\bar{y}}^{2}}-(k_{1}\bar{w}-k_{2}S-k_{2}\bar{\Phi })=0$$

where $\bar{\Phi }=\frac{Br}{{\left(1-\phi \right)}^{5/2}}\left[{\left|\frac{\partial \bar{w}}{\partial \bar{x}}\right|}^{n-1}{\left(\frac{\partial \bar{w}}{\partial \bar{x}}\right)}^{2}+{\left|\frac{\partial \bar{w}}{\partial \bar{y}}\right|}^{n-1}{\left(\frac{\partial \bar{w}}{\partial \bar{y}}\right)}^{2}\right]$,$k_{1}=k_{2}\left(4+S+\frac{D_{h}{}^{2}}{ab}\int_{0}^{a/D_{h}}\int_{0}^{b/D_{h}}\bar{\Phi }d\bar{x}d\bar{y}\right)/{\bar{w}}_{m}$, $k_{2}=k_{f}/k_{eff}$,$Br=mD_{h}/q_{s}\cdot {\left(W/D_{h}\right)}^{n+1}$ denotes the Brinkman number, which gives the measure of the ratio of heat produced by viscous dissipation to the heat transported by molecular conduction. The relevant dimensionless boundary conditions are

(16) $$\partial \bar{T}/\partial \bar{x}{|}_{\bar{x}=0}=0,\partial \bar{T}/\partial \bar{y}{|}_{\bar{y}=0}=0,\partial \bar{T}/\partial \bar{x}{|}_{\bar{x}=b/D_{h}}=k_{f}/k_{eff},\partial \bar{T}/\partial \bar{y}{|}_{\bar{y}=a/D_{h}}=k_{f}/k_{eff},(\mathrm{or}\;\bar{T}{|}_{\bar{y}=a/D_{h}}=0)$$

With Equations (15) and (16), the dimensionless temperature $\bar{T}$ and the bulk mean temperature ${\bar{T}}_{m}=\int_{0}^{a/D_{h}}\int_{0}^{b/D_{h}}\bar{w}\bar{T}d\bar{x}d\bar{y}/\int_{0}^{a/D_{h}}\int_{0}^{b/D_{h}}\bar{w}d\bar{x}d\bar{y}$. As a result, using the expression of Nusselt number $Nu=hD_{h}/k_{eff}$ and the expression of heat transfer coefficient $h=q_{s}/(T_{w}-T_{m})$, the Nusselt number for power-law nanofluid electrokinetic flow denoting the heat transfer rate is obtained as

(17) $$Nu=-\frac{k_{f}}{k_{eff}}\cdot \frac{1}{{\bar{T}}_{m}}.$$

## 3. Solution Methodology

Since the small zeta potential (*ξ* ≤ 0.025 V) is considered, the known Debye–Hückel approximation $\sinh\bar{\psi }\approx \bar{\psi }$ is adopted [5], whereby P–B equation is linearized. Consequently, an analytical electric potential has been acquired by solving Equations (3) and (4) based on the variable separation method

(18) $$\begin{array}{c} \bar{\psi }(\bar{x},\bar{y})=\frac{2D_{h}\bar{\xi }}{a}\sum_{M=1}^{\infty }\frac{{\left(-1\right)}^{M+1}\cos(\mu_{M}\bar{y})\cosh(\sqrt{\mu_{M}^{2}+K^{2}}\bar{x})}{\mu_{M}\cosh(\sqrt{\mu_{M}^{2}+K^{2}}b/D_{h})}\\ +\frac{2D_{h}\bar{\xi }}{b}\sum_{N=1}^{\infty }\frac{{\left(-1\right)}^{N+1}\cos(\sigma_{N}\bar{x})\cosh(\sqrt{\sigma_{N}^{2}+K^{2}}\bar{y})}{\sigma_{N}\cosh(\sqrt{\sigma_{N}^{2}+K^{2}}a/D_{h})} \end{array}$$

where $\mu_{M}=(2M-1)\pi D_{h}/(2a)$,$\sigma_{N}=(2N-1)\pi D_{h}/(2b)$, *M*,*N* = 1,2,3,….

### 3.1. In the Case of Newtonian Nanofluid Flow

For Newtonian nanofluid (*n* = 1), the modified Cauchy momentum equation, i.e., Equation (9) falls into the following simplified form

(19) $$\left(\frac{\partial^{2}\bar{w}}{\partial {\bar{x}}^{2}}+\frac{\partial^{2}\bar{w}}{\partial {\bar{y}}^{2}}\right)-{\left(1-\phi \right)}^{5/2}(\frac{d\bar{p}}{dz}+G_{1}E_{s}\bar{\psi })=0$$

With the boundary conditions expressed as Equation (10), the analytical velocity is obtained based on Green’s function method and the method of variable separation

(20) $$\bar{w}(\bar{x},\bar{y})=-\frac{4D_{h}{}^{2}{\left(1-\phi \right)}^{5/2}}{ab}[\frac{d\bar{p}}{dz}\sum_{N=1}^{\infty }\sum_{M=1}^{\infty }\frac{{\left(-1\right)}^{M+N}\cos(\sigma_{N}^{}\bar{x})\cos(\mu_{M}\bar{y})}{\mu_{M}\sigma_{N}^{}\lambda_{NM}{}^{2}}+G_{1}{\bar{E}}_{s}\sum_{N=1}^{\infty }\sum_{M=1}^{\infty }C_{NM}\frac{\cos(\sigma_{N}^{}\bar{x})\cos(\mu_{M}\bar{y})}{\lambda_{NM}{}^{2}}]$$

where 

$\lambda_{NM}{}^{2}=\mu_{M}{}^{2}+\sigma_{N}^{2}$,$C_{NM}==\frac{{\left(-1\right)}^{N+M}\bar{\xi }}{\sigma_{N}\mu_{M}}\left[{\left(1+\frac{K^{2}+\sigma_{N}{}^{2}}{\mu_{M}{}^{2}}\right)}^{-1}+{\left(1+\frac{K^{2}+\mu_{M}{}^{2}}{\sigma_{N}{}^{2}}\right)}^{-1}\right]$. The specific solution procedure can be found in Appendix A.

Accordingly, the analytical mean velocity on the cross-section area of rectangular microchannel has the following form

(21) $${\bar{w}}_{m}(\bar{x},\bar{y})=-\frac{4D_{h}{}^{2}{\left(1-\phi \right)}^{5/2}}{ab}[\frac{d\bar{p}}{dz}\sum_{N=1}^{\infty }\sum_{M=1}^{\infty }\frac{1}{\mu_{M}{}^{2}\sigma_{N}^{2}\lambda_{NM}{}^{2}}+G_{1}{\bar{E}}_{z}\sum_{N=1}^{\infty }\sum_{M=1}^{\infty }\frac{{\left(-1\right)}^{N+M}C_{NM}}{\mu_{M}\sigma_{N}^{}\lambda_{NM}{}^{2}}]$$

The electrokinetic flow of Newtonian fluid becomes pure PDF when the streaming potential effect is eliminated. As a result, the analytical velocity for the pure PDF of Newtonian fluid can be derived from Equation (20)

(22) $${\bar{w}}_{0}(\bar{x},\bar{y})=-\frac{4D_{h}{}^{2}{\left(1-\phi \right)}^{5/2}}{ab}\frac{d\bar{p}}{dz}\sum_{N=1}^{\infty }\sum_{M=1}^{\infty }\frac{{\left(-1\right)}^{M+N}\cos(\sigma_{N}^{}\bar{x})\cos(\mu_{M}\bar{y})}{\mu_{M}\sigma_{N}^{}\lambda_{NM}{}^{2}}$$

Combining Equations (21) and (22) above with the expression of dimensionless induced electric field strength Equation (11) and yields the explicit form of the induced electric field strength

(23) $${\bar{E}}_{s}=\frac{-\frac{4D_{h}{}^{2}{\left(1-\phi \right)}^{5/2}}{ab}\frac{d\bar{p}}{dz}A_{1}\sum_{N=1}^{\infty }\sum_{M=1}^{\infty }\frac{{\left(-1\right)}^{N+M}C_{NM}}{\mu_{M}\sigma_{N}^{}\lambda_{NM}{}^{2}}}{1+{\left(1-\phi \right)}^{5/2}A_{1}A_{2}\sum_{N=1}^{\infty }\sum_{M=1}^{\infty }\frac{C_{NM}{}^{2}}{\lambda_{NM}{}^{2}}}$$

Based on the method of variable separation and method of constant variation [34,39], the temperature field in the absence of viscous dissipation for Newtonian nanofluid electrokinetic flow through a rectangular microchannel has been firstly obtained as

(24) $$\bar{T}(\bar{x},\bar{y})=\sum_{I=1}^{\infty }\cos(C_{I}\bar{x})[Y_{IG}(\bar{y})+Y_{IP}(\bar{y})]$$

where

(25) $$\begin{array}{c} Y_{IG}(\bar{y})=\frac{8k_{1}D_{h}{}^{3}{\left(1-\phi \right)}^{5/2}\cosh(C_{I}\bar{y})}{ab^{2}R_{I}}[\frac{d\bar{p}}{dz}\sum_{N=1}^{\infty }\sum_{M=1}^{\infty }\frac{{\left(-1\right)}^{M+N}A_{MI}B_{NI}}{\mu_{M}\sigma_{N}^{}\lambda_{NM}{}^{2}}\\ +G_{1}{\bar{E}}_{s}\sum_{N=1}^{\infty }\sum_{M=1}^{\infty }\frac{A_{MI}B_{NI}C_{NM}}{\lambda_{NM}{}^{2}}]+B_{I}\cosh(C_{I}\bar{y})\left(\frac{1}{R_{I}}-\frac{1}{C_{I}}\right) \end{array}$$

(26) $$\begin{array}{c} Y_{IP}(\bar{y})=-\frac{8k_{1}D_{h}{}^{3}{\left(1-\phi \right)}^{5/2}}{ab^{2}}[\frac{d\bar{p}}{dz}\sum_{N=1}^{\infty }\sum_{M=1}^{\infty }\frac{{\left(-1\right)}^{M+N}B_{NI}D_{MI}[\cos(\mu_{M}\bar{y})-\cosh(C_{I}\bar{y})]}{\mu_{M}\sigma_{N}^{}\lambda_{NM}{}^{2}}\\ +G_{1}{\bar{E}}_{s}\sum_{N=1}^{\infty }\sum_{M=1}^{\infty }\frac{B_{NI}C_{NM}D_{MI}[\cos(\mu_{M}\bar{y})-\cosh(C_{I}\bar{y})]}{\lambda_{NM}{}^{2}}]+\frac{B_{I}}{C_{I}{}^{2}}[1-\cosh(C_{I}\bar{y})] \end{array}$$

with $A_{MI}=-\frac{C_{I}}{C_{I}{}^{2}+\mu_{M}{}^{2}}[\cos(\mu_{M}a/D_{h})-\cosh(C_{I}a/D_{h})]$,$B_{I}=2{\left(-1\right)}^{I+1}k_{2}SD_{h}C_{I}/b$,$B_{NI}=\left\{ \begin{array}{l} b/(2D_{h}),\;N=I\\ 0,\;N\neq I \end{array}\right.$,$C_{I}=\sqrt{\left(2I-1)\pi D_{h}/(2b\right)}$,$D_{MI}=-\frac{1}{C_{I}{}^{2}+\mu_{M}{}^{2}}$,$R_{I}=C_{I}\cosh(C_{I}a/D_{h})$. The specific solution procedure is presented in Appendix B.

### 3.2. In the Ccase of Power-Law Nanofluid

When considering power-law nanofluid flow (*n* ≠ 1 and *ϕ* ≠ 0) with streaming potential effect and viscous dissipation, according to the coupling of Equations (9)–(11), (15), and (16), the velocity distribution and induced electric field strength need to be solved to acquire temperature distribution and Nusselt number. Due to the high nonlinearity of modified Cauchy momentum Equations (9) and (10) and energy Equations (15) and (16), high order finite difference methods have been applied to solve the velocity and temperature [34]. The term $\partial /\partial \bar{t}$ is introduced to iteratively solve hydrodynamic field and thermal field from $\partial f/\partial \bar{t}=D_{1}\partial^{2}f/\partial {\bar{x}}^{2}+D_{2}\partial^{2}f/\partial {\bar{y}}^{2}+D_{3}$ where $D_{1}=nm/[\mu_{0}{\left(1-\phi \right)}^{5/2}]{\left(W/D_{h}\right)}^{n-1}{\left|\partial \bar{w}/\partial \bar{x}\right|}^{n-1}$, $D_{2}=nm/[\mu_{0}{\left(1-\phi \right)}^{5/2}]{\left(W/D_{h}\right)}^{n-1}{\left|\partial \bar{w}/\partial \bar{y}\right|}^{n-1}$, $D_{3}=-{\left(1-\phi \right)}^{5/2}(d\bar{p}/dz+G_{1}{\bar{E}}_{s}\bar{\psi })$, and *f* = *w* when solving Equations (9) and (10). The nonlinear coefficients are numerically treated by the compact difference scheme that can be found in our last works [17,34]. When solving Equations (15) and (16), *D*_1_ *=* 1, *D*_2_ *=* 1, $D_{3}=-(k_{1}\bar{w}-k_{2}S-k_{2}\bar{\Phi })$ and f=T. In terms of time variable, the time splitting method is used. In the first half time step, $\partial f/\partial \bar{t}=D_{3}(\bar{x},\bar{y})$ is numerically solved based on the Runge–Kutta method and in the second half time step the Crank–Nicolson method is employed to solve $\partial f/\partial \bar{t}=D_{1}\partial^{2}f/\partial {\bar{x}}^{2}+D_{2}\partial^{2}f/\partial {\bar{y}}^{2}$. The discretization procedure is found in details in [17,34]. A specified criterion *Er* is given to identify that if the velocity is fully developed, i.e., $\left\|f_{}^{l}-f_{}^{l+1}\right\|<Er$ because $\partial \bar{w}/\partial \bar{t}{|}_{\bar{t}\to \infty }=\partial \bar{T}/\partial \bar{t}{|}_{\bar{t}\to \infty }=0$. Eventually, the fully developed numerical velocity and numerical temperature are acquired.

## 4. Method Validation

**Figure 2:**
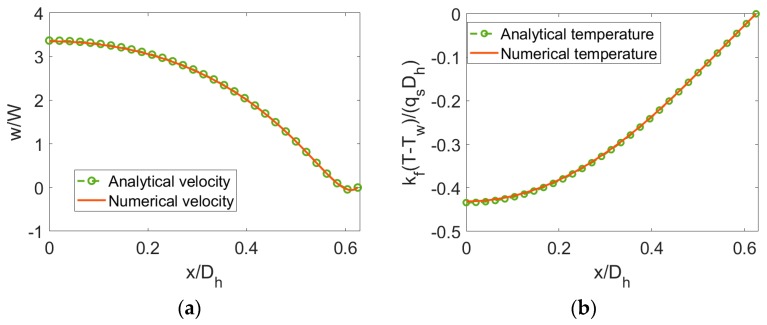
The comparison of numerical solutions with the analytical solutions for (**a**) velocity and (**b**) temperature.

The volumetric domain *Ω* is discretized to a grid system of 101 × 151 (*y* × *x*). A test of grid dependence is conducted and thus the numerical methods are verified. The numerical and analytical results are compared when applying Debye–Hückel approximation and neglecting viscous dissipation. In Figure 2, the numerical velocity profile at $\bar{y}=0$ is compared to the analytical velocity profile obtained from Equation (20) and the numerical temperature profile at $\bar{y}=0$ is compared to the analytical temperature profile obtained from Equation (24) when *K* = 10, *ϕ* = 0.06 and *S* = 3. To render the comparison clearer, only 31 grid points of the numerical solution are plotted in Figure 2. The good agreement indicates that the numerical method proposed above can be applied to solve velocity, temperature, and Nusselt number of power-law nanofluid PDF under streaming potential effect.

## 5. Results and Discussion

For different types of power-law nanofluids, a parametric study for the hydrodynamical and thermal fields is carried out where the influence of flow behavior index *n*, electrokinetic width *K*, volume fraction of nanoparticles *ϕ*, Brinkman number *Br,* and Joule heating parameter *S* is studied. The nanoparticle is regarded as aluminum oxide [31] and the choice of associated physical parameters can be referred to literature [9] and [17]. The typical values are presented in Table 1 below.

**Figure 3:**
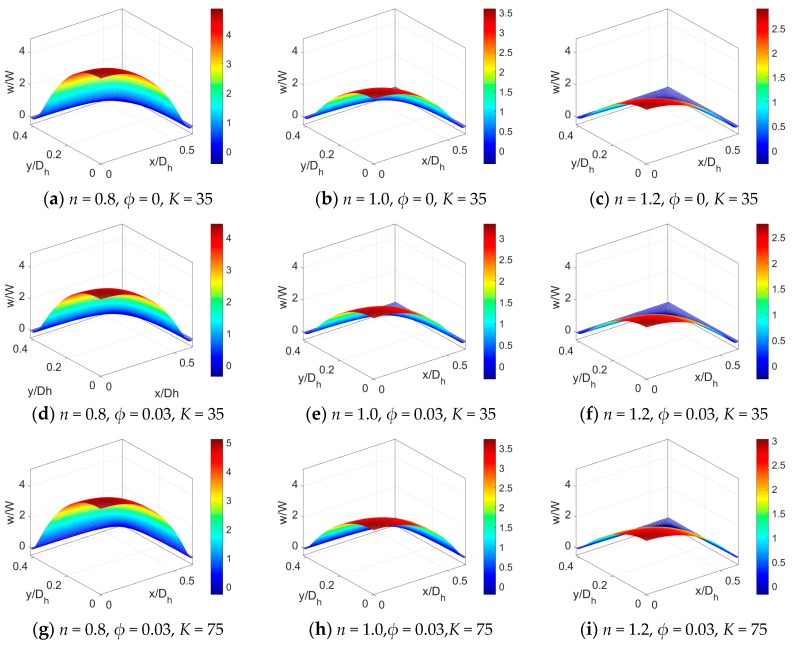
The comparison of velocity distributions across the rectangular microchannel at different flow behavior *n*, different particle volume fraction *ϕ* and different dimensionless electrokinetic width *K.*

The velocity distributions across the rectangular microchannel at different parameters are presented in Figure 3. At first, the velocity distributions of shear thinning, Newtonian, and shear thickening fluids have been respectively presented in Figure 3a–c in the case of *K* = 35. To show the influence of nanoparticle volume fraction, Figure 3d–f provides the velocity distributions of power-law nanofluids when the volume fraction of nanoparticle increases from *ϕ* = 0 to *ϕ* = 0.03 and other parameters remain unchanged. Compared to pure fluids, the addition of nanoparticle increases fluid viscosity, and thus the decrease in velocity for power-law nanofluid is observed, which is more evident for shear thinning base fluid. Further, the streaming potential effect has been investigated through the comparison of Figure 3d–f with Figure 3g–i by increasing the dimensionless electrokinetic width *K* and keeping other parameters the same. To note, a stronger streaming potential is represented by a larger electrokinetic width *K*, since larger K is obtained by increasing the EDL length. It shows that for power-law nanofluids, the less the magnitude of *K*, the more obvious the streaming potential effect on velocity, the more the velocity profile gets retarded, especially in the vicinity of channel walls.

**Figure 4:**
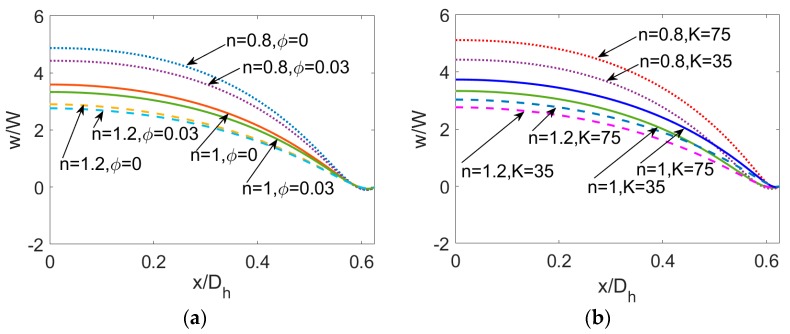
The comparison of velocity profile at $\bar{y}=0$ for different flow behavior *n*, different particle volume fraction *ϕ* and different dimensionless electrokinetic width *K*. (**a**) The influence of *ϕ* when *K* = 35, (**b**) The influence of *K* when *ϕ* = 0.03.

To clearly present the influence of flow behavior index *n* of power-law nanofluid, nanoparticle volume fraction *ϕ* and dimensionless electrokinetic width *K*, the velocity profiles at $\bar{y}=0$ are plotted in Figure 4 where the parameters take the same value as that in Figure 3. It is obvious that no matter what value the particle volume fraction takes, the bulk liquid velocity of PDF decreases when flow behavior index *n* increases. In the limiting case of power-law fluid, the familiar variation of velocity with *n* has been observed in our last work [17]. The streaming potential effect on velocity at a lower value of dimensionless electrokinetic width *K* gets stronger due to the increase in length of EDL, which is more pronounced for shear thinning nanofluid.

**Figure 5:**
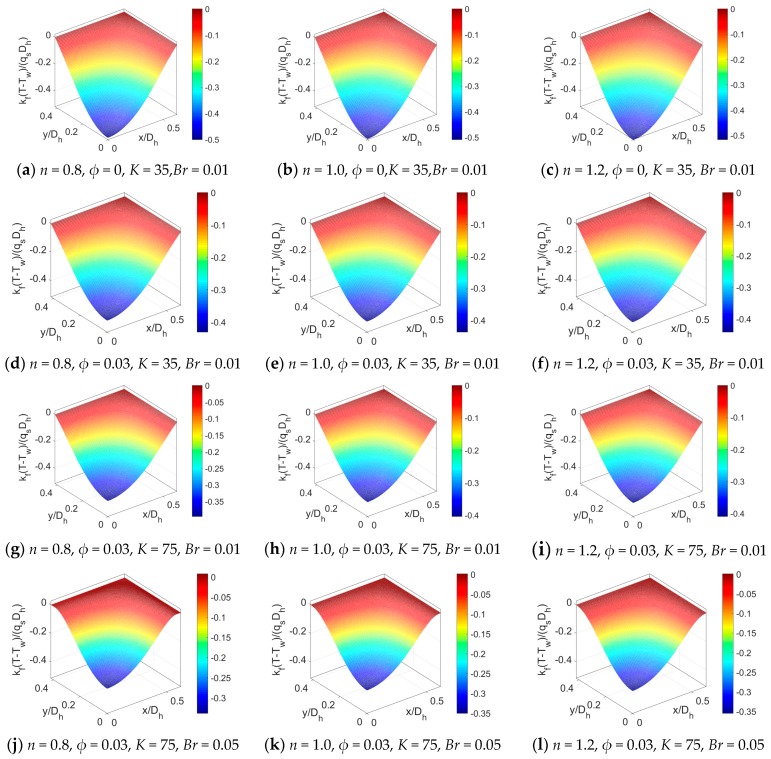
The comparison of temperature distributions across the rectangular microchannel at different flow behavior *n*, different particle volume fraction *ϕ*, different dimensionless electrokinetic width *K* and different Brinkman number *Br*.

The temperature distributions across the rectangular microchannel in the case of different parameters are presented in Figure 5. The temperature distributions for different fluid types, namely, for shear thinning fluid, Newtonian, and shear thickening fluid are compared in Figure 5a–c. It is noted that the temperature distribution is a weak function of flow behavior index *n* for the PDF with streaming potential effect. To study the influence of nanoparticle, the volume fraction of nanoparticle *ϕ* is increased from 0 to 0.03 in Figure 5d–e. The comparison between Figure 5a–c and Figure 5d–e indicates that the temperature difference between the channel wall and the bulk flow is reduced and the bulk mean temperature is enhanced. In Figure 5g–i, the temperature at a larger value of dimensionless electrokinetic width *K* is presented when considering nanofluid. From the comparison of Figure 5d–f with Figure 5g–i, the increment of dimensionless electrokinetic width *K* leads to a weaker streaming potential effect, as a result, the temperature distribution becomes wider and the temperature difference declines. In other words, for electrokinetic flow of power-law nanofluid, the streaming potential effect reduces the temperature in the vicinity of wall rather than the bulk flow, leading to the enlargement in temperature difference between the wall and bulk flow. It means (the presence of) a stronger streaming potential relatively promotes the conversion of mechanical energy to thermal energy near the channel wall. Since the Brinkman number *Br* is the measurement of heat produced by viscous dissipation to the heat transported by molecular conduction, in Figure 5j–l, the Brinkman number is increased from 0.01 to 0.05 and other parameters remain unchanged. The influence of *Br* is studied by comparing Figure 5g–i with Figure 5j–l. The increase in *Br* not only reduces temperature difference, but also retards the temperature distribution near the channel wall.

**Figure 6:**
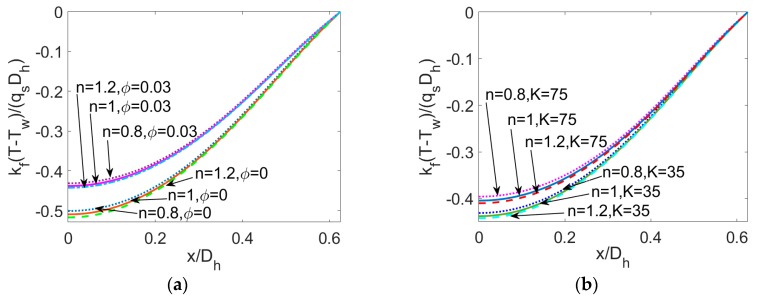

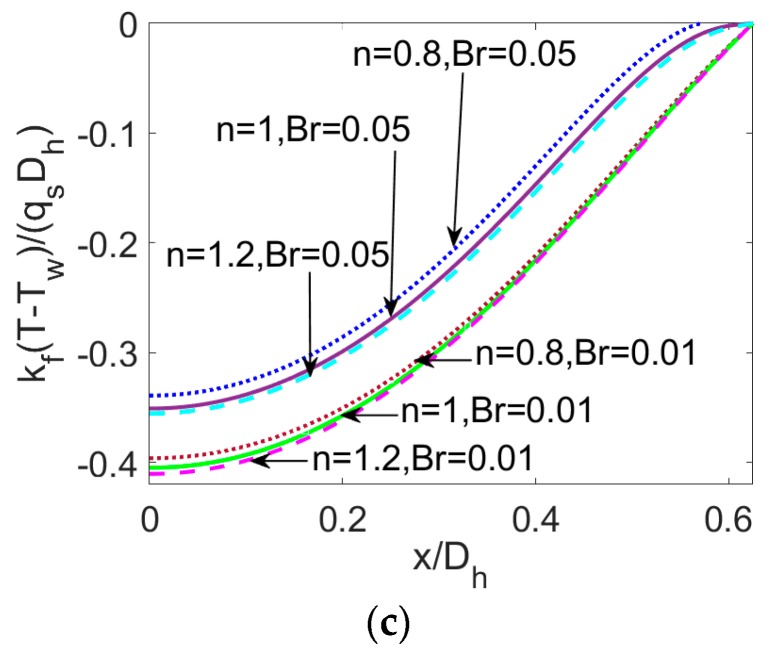
The comparison of temperature profiles at $\bar{y}=0$ for different flow behavior *n*, different particle volume fraction *ϕ*, different dimensionless electrokinetic width *K* and different Brinkman number (*S* = 0.5). (**a**) The influence of *ϕ* when *K* = 35, *Br* = 0.01. (**b**) The influence of *K* when *ϕ* = 0.03, *Br* = 0.01. (**c**) The influence of *K* when *K* = 75, *ϕ* = 0.03.

To provide a detailed insight to the temperature variation at different parameters, the temperature profiles at $\bar{y}=0$ for different parameters are plotted together in Figure 6 where the parameters take the same value as that in Figure 5. The variation of temperature with the nanoparticle volume fraction *ϕ*, electrokinetic width *K,* and Brinkman number *Br* for three types of nanofluid are plotted in Figure 6a–c, respectively. It can be clearly seen that even for power-law nanofluid, the variation tendency of temperature profile with the electrokinetic width *K* and nanoparticle volume fraction *ϕ* is consistent with that in temperature profiles in [20]. The influence of flow behavior index *n*, namely the change in constitutive behavior of base fluid, is slight compared to the influence of other parameters.

**Figure 7:**
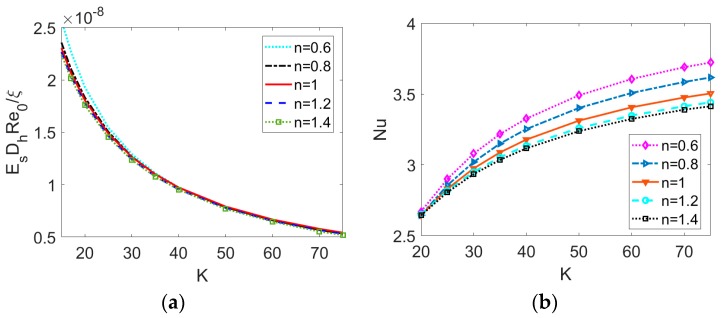
The effect of dimensionless electrokinetic width on (**a**) the induced electric field strength and (**b**) the Nusselt number for different types of base fluid when *ϕ* = 0.03, *Br* = 0.01, *S* = 0.5.

To obtain a thorough understanding on streaming potential effect in the thermal transport characteristics for the PDF of power-law nanofluid, besides the temperature distribution, the strength of induced electric field and Nusselt number denoting the heat transfer rate are presented at different base fluid type and EDL length represented by *K* in Figure 7. From Figure 7a, the induced electric field strength is subject to a weak relation with the fluid type, which shows a decreasing trend with the dimensionless electrokinetic width *K*. It means that no matter what type of nanofluid is considered, the length of EDL is responsible for the streaming potential effect. As shown in Figure 7b, the Nusselt number *Nu* (the heat transfer rate) rises in the case of larger value of *K* and smaller value of *n*. It is due to the fact that the increase in temperature of bulk fluid caused by larger *K* will intensify the heat transfer performance. This is also consistent with the enhanced bulk temperature observed in Figure 5g–i. The variation tendency of Nusselt number Nu with *K* and *n* is in line with the result in the case of cylindrical microcapillary [20]. In comparison with the Newtonian and shear thickening nanofluid, the shear thinning nanofluid is sensitive to the temperature variation and thus an intensified heat transfer performance is observed.

**Figure 8:**
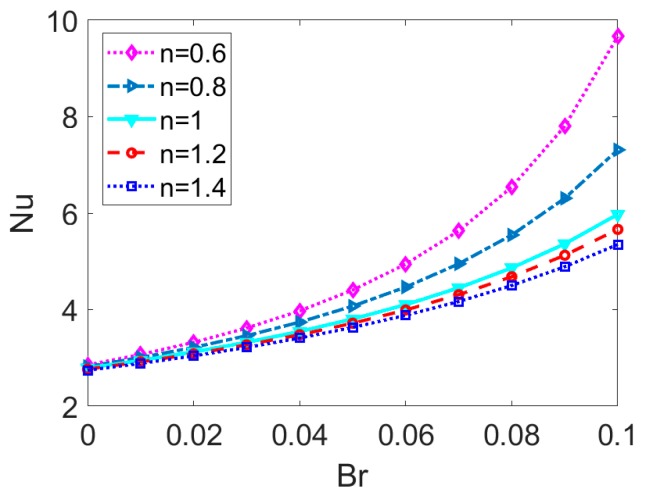
The effect of Brinkman number on Nusselt number for different types of base fluid when *ϕ* = 0.03, *K* = 35, *S* = 1.

The variation of Nusselt number *Nu* with Brinkman number *Br* for different base fluid type is presented in Figure 8. The Nusselt number increases with *Br* and the increasing rates gets larger for shear thinning nanofluid, compared to the Newtonian and shear thickening nanofluid. It can be attributed to the fact that the viscous dissipation term represented by $\bar{\Phi }$ in Equation (15) is augmented owing to the larger value of *Br*, leading to the reduction of temperature difference between channel wall and bulk liquid and meaning that the heat transfer performance is intensified. Furthermore, the viscous dissipation effect plays a considerable role in the case of shear thinning nanofluid. This is because the shear thinning feature made a bigger contribution to the viscous dissipation term represented by $\bar{\Phi }$ and thus leads to the larger value of Nusselt number *Nu*. For Newtonian fluid, the variation of *Nu* with *Br* is in line with the results in [22].

**Figure 9:**
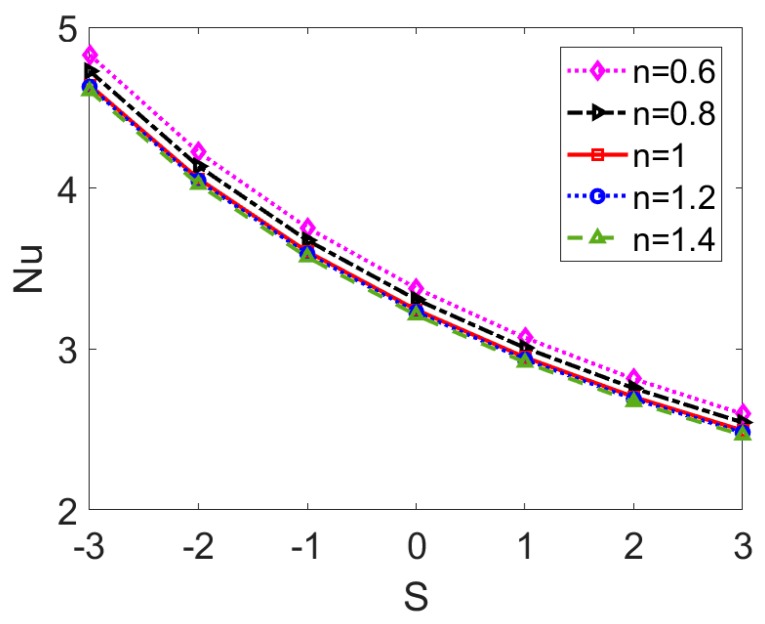
The effect of Joule heating parameter on Nusselt number for different types of base fluid when *ϕ* = 0.03, *Br* = 0.01, *K* = 35.

To show the Joule heating effect on the thermal behavior of electrokinetic flow, the variation of Nusselt number *Nu* with the Joule heating parameter *S* at different base fluid type is provided in Figure 9. The case of S < 0 indicates outward heat flux, namely the surface cooling effect and the case of S > 0 means the surface heating effect. Joule heating effect tends to reduce the Nusselt number *Nu* and the heat transfer rate. Therefore, it reveals that the enhanced Joule heating effect denoted by the increased *S* raises the bulk temperature and the heat transfer performance is reduced consequently. In the case of Newtonian nanofluid, this is consistent with the prediction in [32]. The decreasing rate with Joule heating parameter *S* shows little change for different nanofluid type. This is because the the Joule heating term in Equation (15) is independent of flow behavior index *n*.

**Figure 10:**
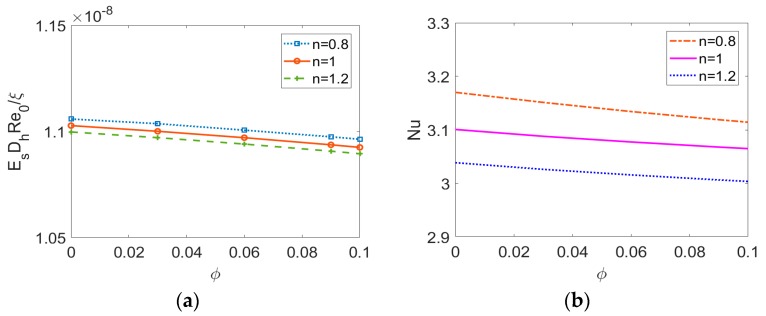
The effect of volume fraction of nanoparticle on (**a**) the induced electric field strength and (**b**) Nusselt number for different types of base fluid when *K* = 35, *Br* = 0.01, *S* = 0.5.

To provide a deep insight into the influence of nanoparticles on electrokinetic flow with streaming potential effect, the induced electric field strength and the Nusselt number are plotted as a function of the volume fraction of nanoparticles, respectively in Figure 10a,b. As shown in Figure 10a, regardless of the base fluid type, the variation of induced electric field strength with the nanoparticle volume fraction *ϕ* is quite slight, and thus it means that the induced electric field is a weak function of *ϕ*. In addition, Figure 10b shows that the nanoparticle volume fraction *ϕ* enhances the bulk mean temperature as predicted by Figure 5d–f, however, which leads to a slight abatement in *Nu*, thereby resulting in the deterioration of the heat transfer rate. It is due to the fact that the decrease of *k_f_*/*k_eff_* in the expression of *Nu* namely Equation (17) caused by the increase in *ϕ*, outweighs the increase of $-1/{\bar{T}}_{m}$, no matter what type of nanofluid is considered. Therefore, one should have a second thought on choosing nanofluid as an approach to improve heat transfer performance.

## 6. Conclusions

When considering the effect of streaming potential, the induced electric potential, velocity, temperature, and heat transfer rate for power-law nanofluid flow are evaluated by solving the P–B equation, the modified Cauchy momentum equation and the energy equation with viscous dissipation effect. The analytical solutions for Newtonian nanofluid flow have been derived based on the method of variable separation and Green’s function method and numerical solutions for power-law nanofluid flow have been developed by applying the implicit finite difference schemes. Proceeding from the evaluation above, thermal transport characteristics are investigated by studying the combined effects of the streaming potential, nanoparticle, fluid type, viscous dissipation, and Joule heating on temperature distribution and heat transfer rate. The following conclusions are drawn:

- For electrokinetic flow of power-law nanofluid, the streaming potential effect not only reduces and retards velocity distribution, but also narrows temperature difference between the bulk flow and channel wall, which in further reduces the Nusselt number. Thus, when considering the streaming potential effect on PDF in microchannels, increasing the electrokinetic width *K* is an effective approach to improve heat transfer performance of PDF.
- The bulk mean temperature rises as the volume fraction of nanoparticle *ϕ* increases no matter what fluid type is considered. However, a slight decrease of Nusselt number *Nu* with *ϕ* is observed and thus one should have a second thought when adding nanoparticles to liquid to enhance the heating transfer rate.
- Regarding the nanofluid type, it is notable that temperature distribution is a weak function of flow behavior index *n*. Compared to the Newtonian nanofluid and especially the shear thickening nanofluid, the shear thinning nanofluid exhibits greater heat transfer rate, indicating it to be more sensitive to the introduction of nanoparticles, the effects of streaming potential, and viscous dissipation. Therefore, to obtain higher heat transfer rate in engineering application, the working liquid can be chosen as shear thinning power-law nanofluid. Moreover, one should carefully consider the heat transfer characteristics when treating biofluids and other liquids with long chain molecules as Newtonian fluids.
- When the Brinkman number *Br* is augmented, the temperature distribution especially in the vicinity of channel wall increases and *Nu* is enhanced correspondingly. It reveals that the viscous dissipation effect plays a part on both temperature profile and Nusselt number, which is more pronounced in the case of shear thinning nanofluid. Therefore, the consideration of viscous dissipation for non-Newtonian fluids is worth the discussion above.
- The Nusselt number *Nu* shows a decreasing trend with Joule heating parameter *S*. The evident difference of *Nu* with and without consideration of Joule heating effect indicates that the Joule heating needs to be carefully considered when studying the heat transfer characteristics in electrokinetic flow of power-law nanofluid.

## Figures and Tables

**Table 1:** Typical values of the parameters.

Parameters (notation)	Value (unit)
The permittivity in vacuum *ε*	8.85 × 10^−12^ C·V^−1^·m^−1^
Boltzmann constant *k_B_*	1.38 × 10^−23^ J·K^−1^
Absolute temperature *T_a_*	293 K
Elementary charge *e*	1.6 × 10^−19^ C
Half channel height *a*	1 × 10^−6^ m
Half channel width *b*	1.5 × 10^−6^ m
Total electrical conductivity *σ*	1.2639 × 10^−7^ S·m^−1^
Flow consistency index of power-law fluid *m*	9 × 10^−4^ N·m*^−^*^2^·s*^n^*
Viscosity of Newtonian fluid *μ*_0_	9 × 10^−4^ N·m^−2^·s
Zeta potential *ξ*	0.025 V
Thermal conductivity of the solid nanoparticle *k_s_*	40 W·m^−1^·K^−1^
Thermal conductivity of the base fluid *k_f_*	0.618 W·m^−1^·K^−1^
The pressure gradient d*p*/d*z*	*−*1 × 10^4^ Pa
The relative permittivity *ε_0_*	80
Valence of ions *z_v_*	1
Electrokinetic width *K*	15–75
Flow behavior index *n*	0.6–1.4
Nanoparticle volume fraction *ϕ*	0.0–0.1
Joule heating Parameter *S*	*−*3 – 3
Brinkman number *Br*	0–0.1
Ratio of nanolayer thickness to original particle radius *ω*	1.1

## Appendix A

To solve the momentum equation for Newtonian nanofluid given as Equation (19) and the corresponding boundary conditions (10), the Green’s function $G(\bar{x},\bar{y},\bar{t};\zeta ,\eta ,\upsilon )$ for the dimensionless transient velocity $\bar{w}(\bar{x},\bar{y},\bar{t})$ is introduced and thus the following partial differential equation is presented

(A1) $$\frac{\partial G}{\partial \bar{t}}=\frac{\partial^{2}G}{\partial {\bar{x}}^{2}}+\frac{\partial^{2}G}{\partial {\bar{y}}^{2}}+\delta (\bar{x}-\varsigma )\delta (\bar{y}-\eta )(\bar{t}-\upsilon )$$

(A2) $$G{|}_{\bar{t}=0}=0,\;\partial G/\partial \bar{x}{|}_{\bar{x}=0}=0\;,\;\partial G/\partial \bar{y}{|}_{\bar{y}=0}=0,\;G{|}_{\bar{x}=b/D_{h}}=0,G{|}_{\bar{y}=a/D_{h}}=0$$

Since a fully developed flow is considered here, namely, $\partial \bar{w}/\partial \bar{t}{|}_{\bar{t}\to \infty }=0$, the fully developed velocity $\bar{w}(\bar{x},\bar{y})$ automatically satisfies

(A3) $$\bar{w}(\bar{x},\bar{y})=\underset{\bar{t}\to \infty }{\lim}\int_{\upsilon =0}^{\bar{t}}G(\bar{x},\bar{y},\bar{t};\varsigma ,\eta ,\upsilon )*[-{\left(1-\phi \right)}^{5/2}(\frac{d\bar{p}}{dz}+G_{1}{\bar{E}}_{s}\bar{\psi })]d\upsilon$$

based on the Green’s function method. To derive the explicit expression of $\bar{w}(\bar{x},\bar{y})$, according to the method of variable separation, the following homogeneous version of Equations (A1) and (A2) needs to be solved,

(A4) $$\frac{\partial G^{\prime }}{\partial \bar{t}}=\frac{\partial^{2}G^{\prime }}{\partial {\bar{x}}^{2}}+\frac{\partial^{2}G^{\prime }}{\partial {\bar{y}}^{2}}$$

(A5) $$G^{\prime }{|}_{\bar{t}=0}=\delta (\bar{x}-\varsigma )\delta (\bar{y}-\eta )(\bar{t}-\upsilon ),\;\partial G^{\prime }/\partial \bar{x}{|}_{\bar{x}=0}=0,\;\partial G^{\prime }/\partial \bar{y}{|}_{\bar{y}=0}=0,\;G^{\prime }{|}_{\bar{x}=b/D_{h}}=0,\;G^{\prime }{|}_{\bar{y}=a/D_{h}}=0$$

with the condition $G(\bar{x},\bar{y},\bar{t};\varsigma ,\eta ,\upsilon )=\int_{\upsilon =0}^{\bar{t}}G^{\prime }(\bar{x},\bar{y},\bar{t};\varsigma ,\eta ,\upsilon )d\upsilon$. Applying the superposition method to Equations (A4) and (A5), the Green function is obtained as

(A6) $$G(\bar{x},\bar{y},\bar{t};\varsigma ,\eta ,\upsilon )=\frac{4D_{h}{}^{2}}{ab}\sum_{M=1}^{\infty }\sum_{N=1}^{\infty }e^{-\lambda_{NM}^{2}(\bar{t}-\upsilon )}\cos(\sigma_{N}\varsigma )\cos(\mu_{M}\eta )\cos(\sigma_{N}\bar{x})\cos(\mu_{M}\bar{y})$$

where *λ_NM_*=(*σ_N_*+*μ_M_*)^1/2^. As a result, replacing Equation (A6) into Equation (A3), and combining with the analytical electric potential $\bar{\psi }$ presented as Equation (18), one can acquire the dimensionless fully developed velocity expressed as Equation (20).

## Appendix B

In the limiting case that the viscous dissipation is neglected, the energy equation becomes

(A7) $$\frac{\partial^{2}\bar{T}}{\partial {\bar{x}}^{2}}+\frac{\partial^{2}\bar{T}}{\partial {\bar{y}}^{2}}=k_{1}\bar{w}-k_{2}S$$

where $k_{1}=k_{2}\left(4+S\right)/{\bar{w}}_{m}$, and $k_{2}=k_{f}/k_{eff}$. According to the method of variable separation [39], the temperature field can be expressed as

(A8) $$\bar{T}(\bar{x},\bar{y})=\sum_{I=1}^{\infty }\cos(C_{I}\bar{x})Y_{I}(\bar{y}),\;\mathrm{with}\;C_{I}=\sqrt{\left(2I-1)\pi D_{h}/(2b\right)}$$

To obtain the explicit form of dimensionless temperature $\bar{T}$, $Y_{I}(\bar{y})$ is solved at first as follows.

(i) With the substitution of Equation (A8) into Equation (A7), one has

(A9) $$\left[\frac{d^{2}Y_{I}(\bar{y})}{d{\bar{y}}^{2}}-C_{I}{}^{2}Y_{I}(\bar{y})\right]=S_{I}(\bar{y})$$

where the source term $S_{I}(\bar{y})$ satisfies $\sum_{I=1}^{\infty }\cos(C_{I}\bar{x})S_{I}(\bar{y})=k_{1}\bar{w}-k_{2}S$. Based on the orthogonal property of Fourier series and the expression of dimensionless velocity $\bar{w}$ given as Equation (20), we have

(A10) $$S_{I}(\bar{y})=-\frac{8k_{1}D_{h}{}^{3}}{ab^{2}}[\frac{d\bar{p}}{dz}\sum_{N=1}^{\infty }\sum_{M=1}^{\infty }\frac{{\left(-1\right)}^{M+N}\cos(\mu_{M}\bar{y})B_{NI}}{\mu_{M}\sigma_{N}^{}\lambda_{NM}{}^{2}}+G_{1}{\bar{E}}_{s}\sum_{N=1}^{\infty }\sum_{M=1}^{\infty }C_{NM}\frac{\cos(\mu_{M}\bar{y})B_{NI}}{\lambda_{NM}{}^{2}}]-B_{I}$$

where $B_{NI}=\left\{ \begin{array}{ll} b/(2D_{h}), & N=I\\ 0 & N\neq I \end{array}\right.$.

(ii) Applying the method of constant variation to Equation (A9) produces the following solution form of $Y_{I}(\bar{y})$

(A11) $$Y_{I}(\bar{y})=Y_{IG}(\bar{y})+Y_{IP}(\bar{y})$$

in which $Y_{IG}(\bar{y})=H_{1}e^{C_{I}\bar{y}}+H_{2}e^{-C_{I}\bar{y}}$ and $Y_{IP}(\bar{y})=\frac{1}{C_{I}}\int_{0}^{\bar{y}}S_{I}(y)\sinh[C_{I}(\bar{y}-p)]dp$ with *p* is a temporary variable. The coefficient *H*_1_ and *H*_2_ are obtained by using Equation (A10) and the boundary condition presented as Equation (16). Consequently, the further calculation yields $Y_{I}(\bar{y})$ as presented in Equation (24).
